# Relation between semen oxidative reduction potential in initial semen examination and IVF outcomes

**DOI:** 10.1002/rmb2.12501

**Published:** 2023-01-28

**Authors:** Kazuhisa Tomita, Kankanam Gamage Sanath Udayanga, Manabu Satoh, Shu Hashimoto, Yoshiharu Morimoto

**Affiliations:** ^1^ HORAC Grand Front Osaka Clinic Osaka Japan; ^2^ IVF Namba Clinic Osaka Japan; ^3^ Osaka Metropolitan University Graduate School of Medicine Osaka Japan

**Keywords:** in vitro fertilization outcome, oxidative reduction potential, oxidative stress, semen

## Abstract

**Purpose:**

The MiOXSYS system is a new technique to analyze the semen oxidative reduction potential (ORP) that may use to classify the level of sperm DNA integrity. It does not clearly explain how the semen ORP values could help to change the IVF outcomes. We have analyzed correlations between semen ORP value and the IVF results.

**Methods:**

Four hundred and thirty couples were enrolled. The male counterparts were divided into two groups according to their semen ORP values and compared the fertilization rate, cell cleavage rate, and embryo quality, following the IVF procedures. The relations between ORP values and the clinical pregnancy, live birth, and abortion rates were analyzed.

**Results:**

The ORP values show negative and positive correlations with some conventional semen parameters. The fertilization and the cleavage rate did not show any differences in those two groups, but the transferable embryo rate was significantly high in patients with high semen ORP. However, the patients with high ORP show a tendency to lower clinical pregnancy with a low abortion rate compared to the low ORP group.

**Conclusion:**

The main purpose of measuring the ORP value in semen is still questionable and shows controversial results.

## 1 INTRODUCTION

There have been various kinds of semen analytical and selection strategies in clinical practice, such as selection by morphology, sperm motility, membrane characteristics, sperm DNA fragmentation, etc., which are being decided according to the clinical or patients' demands.^1^, ^2^ All these widely practiced methods have been subjected to considerable debates about the ability to accurately predict in vivo and in vitro embryo development.^3^

Among the men with infertility issues, a higher percentage of sperm DNA fragmentation can be found compared to the fertile men, which strongly suggests that sperm DNA fragmentation may significantly affect male fertility.^4^ The stability of sperm DNA integrity has been shown to be an essential factor that affects the functional competence of the sperm which associates with fertilization, embryo quality, pregnancy, miscarriage, and live birth capacity.^5^ Advanced sperm functional assays such as seminal sperm DNA fragmentation (SDF) analysis and oxidative stress (OS) analysis got into the male infertility laboratory practices which so far are showing promising results in sperm quality analysis.^6^, ^7^, ^8^ The sperm chromatin structure assay (SCSA), the sperm chromatin dispersion (SCD) test, the terminal deoxynucleotidyl transferase‐mediated deoxyuridine triphosphate nick‐end labeling (TUNEL) assay and the single cell gel electrophoresis (Comet) assay are the common methods that are utilized for the assessment of sperm DNA fragmentation.^9^ With regard to the way of sperm DNA fragmentations is occurred, the semen OS has a significant impact not only on DNA integrity but also on epigenetic reprogramming, which may be harmful to the paternal genetic and epigenetic contribution to the development of embryos.^10^, ^11^ The sperms are vulnerable to the destructive forces of ROS as their cell membrane contains relatively high amounts of unsaturated fatty acids that can be undergone oxidative degradation by reactive oxygen species (ROS). Moreover, sperm is unable to neutralize those destructive ROS, as it contains a low amount of scavenging enzymes which leads to exceeding the tolerable level of ROS and total cellular damages.^12^, ^13^, ^14^, ^15^, ^16^ Thus, the level of OS, amount of ROS, and level of sperm DNA fragmentation are all associated factors, and measuring individual or several factors may explain a clear predictive value of the sperm condition related to IVF outcomes.

The OS of biological samples including semen is analyzed by using either a chemiluminescence assay to measure the level of ROS or an electrochemical assay to measure oxidation–reduction potential (ORP),^11^ which provides an overview of the redox system through an assessment of the net balance between oxidants and reductants.^7^ As a biomarker for oxidative stress, ORP has been shown to significantly correlate with semen quality, the severity of the male reproductive illness, and other trauma conditions.^7^, ^11^, ^17^, ^18^ Recently, a novel and simple technology, the MiOXSYS system have been introduced which could rapidly measure ORP in biological samples including semen samples. The MiOXSYS system can measure oxidative stress and static ORP (sORP) at the same time by evaluating the existing balance between total oxidants and reductants in a sample. And it has been confirmed that a cut‐off value of semen sORP can be used as the predictive value of sperm quality determination.^19^, ^20^ Moreover, most recent studies show that infertile sperm samples show a significant positive correlation with ORP and SDF levels compared with fertile controls.^7^, ^10^, ^11^, ^13^ Although many clinics have started to measure sORP using the MiOXSYS system in their patients to determine the quality of the sperm in the initial semen examination. So far the relation between ORP values and IVF outcomes have investigated, and it is now required to explain the prominence of this ORP value to select an IVF treatment strategy.

In this study, we have compared two patient groups who have higher or lower semen sORP values to assess the relationship between the semen ORP value and their IVF outcomes.

## 2 MATERIALS AND METHODS

### 2.1 Patients

The present study was approved by our clinic's Internal Ethical Review Board, and after extensive proper explanation, appropriately signed informed consent was obtained from all the patients by means of opt‐out. Four hundred thirty infertile couples have been enrolled and performed semen examinations in HORAC Grand Front Osaka Clinic from 2017 to 2019 for this clinical experiment. All patients' semen samples were analyzed for oxidative reduction potential at the time of their general sperm parameter analysis. The male patients who are under the following conditions were excluded; azoospermia, antioxidant therapy, and occupational chemical/radiation exposure. Regarding female background, the partners who were diagnosed with symptoms, such as tubal factor, endometriosis, thyroid dysfunction, immunological infertility, recurrent pregnancy loss, hyperprolactinemia, ovarian factor, uterine fibroid, polycystic ovary syndrome, uterine adenomyosis, and secondary infertility are included (Table A1).

### 2.2 Measurement of oxidative reduction potential

**FIGURE A1:**
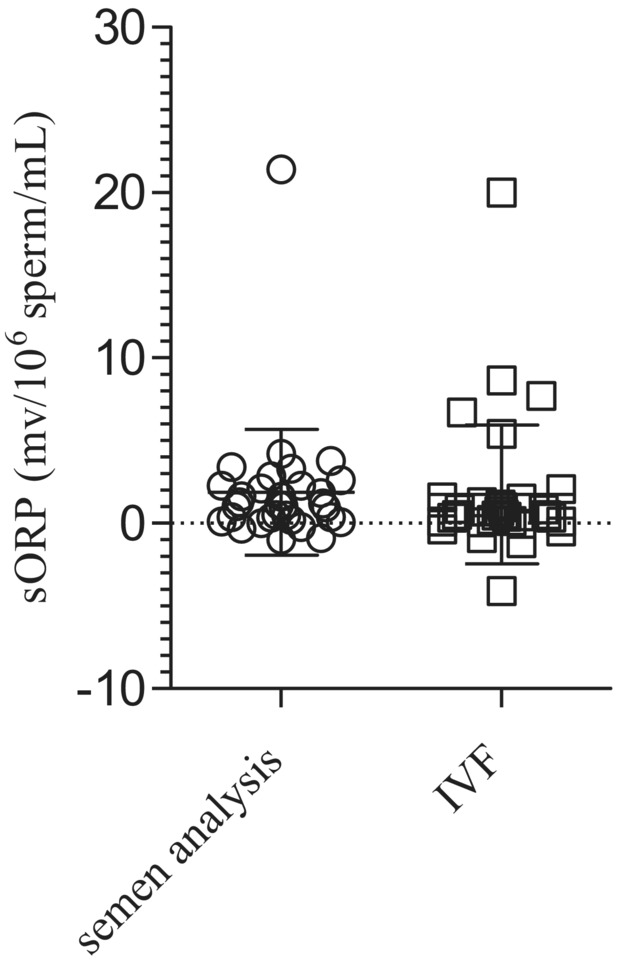
The comparison of static oxidation‐reduction potential (sORP) value between initial semen analysis and IVF. Thirty‐two patients were randomly selected, and compared the sORP value between initial semen analysis and semen for IVF. The circle and square dot indicated the raw data. The bar center and both ends showed the mean value and SD. Statistical significance was analyzed by paired *t*‐test.

Ejaculated semen was liquified at room temperature for 30 min. The analysis of the conventional semen parameter was performed using Macklar chamber® (SEFI‐MEDICAL INSTRUMENT).^21^ The sperm count, motility, and morphology were assessed under 200 times magnification on light microscopy. To analyze semen ORP, MiOXSYS® system was used as previously described in Agarwal et al.^22^ Briefly, 30 μl of liquified semen sample was loaded on the sensor chip and read with MiOXSYS® analyzer. After 2 min, the raw value of ORP was obtained. The raw value was normalized by total sperm concentration (sORP). The relation between sORP and conventional semen parameters or IVF outcomes was investigated. To confirm whether the sORP level has been changed in semen that was used for IVF than the initial analysis, we have re‐analyzed and compared the sORP level in semen before IVF procedures in 32 patients (Figure A1). In this study, according to previous reports and the user guidance of the MiOXSYS system, 1.38 mv/10^6^/ml is the cut‐off value for semen sORP to distinguish the normal and abnormal sperms and 1.41 mv/10^6^/ml is the value that has been used as the cut‐off value to categorize as the infertile or fertile sperms.^18^, ^19^, ^23^, ^24^ The patients who showed higher sORP than 1.38 mv/10^6^/ml were inclined to have higher sperm DNA fragmentations and low fertilization rates, regarded as male infertility.^6^, ^18^, ^24^ We have used both 1.38 or 1.41 (mv/10^6^ sperm/ml) sORP values and compared the quality of sperm according to the traditional semen parameters and further analyzed the fertilization, embryo development, embryo quality, and pregnancy outcomes.

### 2.3 Semen preparation for IVF

To obtain sperm with normal morphology and high motility, semen was washed by a combination of density gradient centrifugation and swim‐up methods. Briefly, liquefied original semen was overlain on the discontinuous concentration of silica particle suspension, and centrifugated at 300 *g* for 20 min. Then, the supernatant was removed, and the sperm sample was washed with a general culture medium of 300 g for 10 min. After washing, the swim‐up method was performed for 15 min or 30 min (ICSI or conventional IVF, respectively).

### 2.4 Ovarian stimulation

The woman received gonadotropin rereleasing hormone (GnRH) agonist protocol treatment or flexible GnRH antagonist protocol treatment as controlled ovarian stimulation as previously described.^25^ Ovarian stimulation was performed by using clomiphene citrate, human menopausal gonadotropin, or recombinant follicle‐stimulating hormone. The average size of some follicles reached 18 mm in diameter, then a maturation trigger was administrated and oocyte retrieval was performed in 36 h.

### 2.5 Assessment embryology

Oocytes were inseminated by ICSI or conventional IVF. The normal fertilization rate was assessed on the next day (day 1). The morphological grade or morphokinetics was evaluated on day 3. Morphological grading was performed by Veeck criteria. The embryos with more than five blastomeres and better than G3 on Veeck criteria were decided as transferable. All embryos were cultured in an incubator equipped with the time‐lapse imaging system, and morphokinetics was recorded every 30 min (iBis; ASTEC). The embryo developed with no direct cleavage until the second cleavage was decided as a normally cleaved embryo.^25^ The rates of fertilization, transferability, and normal cleavage embryo were compared between high and low sORP groups. The investigation of the relation between fertilization and sORP was performed in ICSI cases separately.

### 2.6 Embryo transfer

Single embryo transfer was carried out in 145 couples out of 430 couples. To avoid ovarian hyperstimulation syndrome, frozen single embryo transfer was performed in 83 cycles (57.2%). Clinical pregnancy (CP) was determined by the detection of an intrauterine gestational sac (GS) by transvaginal ultrasound around 3 weeks after embryo transfer as previously described.^26^ Abortion was confirmed if GS was not observed after CP. The relation between CP/live birth/abortion and sORP was analyzed by logistic regression analysis.

### 2.7 Statistical analysis

The correlation between semen parameters and sORP were analyzed by single regression analysis. and regarding embryology, the difference between the two groups was analyzed by the *t*‐test. In addition, the relation between CP/live birth/abortion and sORP was analyzed by logistic regression analysis. As confounder, wife and husband's age were substituted for logistic regression analysis. Data were presented by mean ± standard deviation (SD). Statistical analysis was performed by Statview version 5 (SAS Institute Inc.), and *p* < 0.05 was considered to be significant.

## 3 RESULTS

### 3.1 Patient's semen sample characteristics

The characteristics of conventional semen analysis and sORP of male infertility patients were shown in Table 1 (*n* = 430). All the patients were carefully selected at their first visit as described in the material and the methods. All the semen samples were tested for the general semen parameters (abstinence, total sperm concentration, motility, morphological abnormality, WBC, volume, and pH).

**TABLE 1:** Patient's background.

	Average value	SD
Female age	37.3	4.9
Male age	38.7	6.2
Abstinence (day)	4.1	5.1
Total sperm concentration (10^6^ cell/ml)	6.3	58.7
Total motile sperm count (10^6^ cell)	100.7	119.2
Motility (%)	53.8	19
Abnormality (%)	36.1	14.5
Volume (ml)	2.7	2.7
WBC (10^5^ cell/ml)	5.8	16.9
pH	7.6	0.2
sORP (mV/10^6^ sperm/ml)	3.4	32.0

*Note*: Each data were expressed by mean and SD.

Abbreviations: sORP, static oxidation‐reduction potential; WBC, white blood cell concentration.

### 3.2 The correlations between sORP and semen characteristics

**FIGURE 1:**
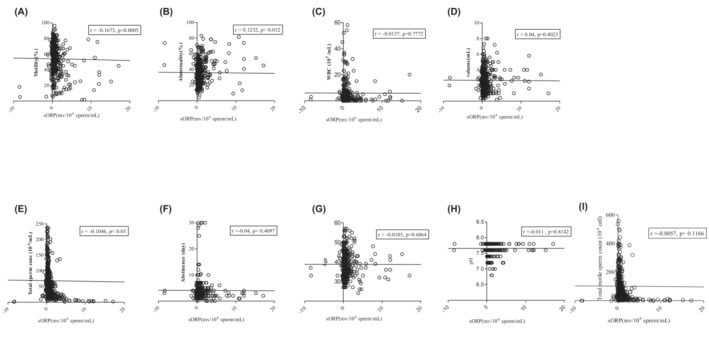
The correlation between static oxidative reduction potential (sORP) and conventional semen parameters. The data show the correlation between sOPR and (A) motility (%), (B) abnormality (%), (C) white blood concentration (WBC) (10^5^ cell/ml), (D) volume (ml), (E) total sperm concentration (10^6^ cell/ml), (F) abstinence (day), (G) age, (H) ph, and (I) total motile sperm count (10^6^ cell). All data were analyzed by single regression analysis.

We assessed whether there was a direct correlation between sORP and other traditional semen factors from 430 male patients. The relationship between sORP and the conventional sperm analytical parameters was analyzed using the MiOXSYS system (Figure 1). There was no significant correlation between the patient's age, the concentration of white blood cells, the total volume of the sample, total motile sperm count, and the abstinence with the semen sORP. Further confirming the previous reports, there was a significant positive correlation between sperm morphology and (*r* = 0.12, *p* < 0.05), significant negative correlations with both sperm concentration and motility with semen sORP (*r* = −0.11, *p* < 0.05; *r* = −0.17, *p* < 0.001). Further, on the day of IVF, the sORP was reanalyzed and compared with the initial sORP in 32 patients. And the individual semen ORP level is not radically altered over the time period between the initial analysis and at the time of IVF (Figure A1). This result suggested that the value of sORP in the initial semen test reflects the value in semen for IVF.

### 3.3 No correlation can be found between sORP and embryology

Then, as the next step of these sORP data, we sought to elucidate the relationship between the semen sORP and fertilization capacity, embryo development, and the pregnancy outcome irrespective of the method of insemination.

This analysis included 393 out of 430 patients who performed a fresh IVF cycle without any antioxidant oral supplementation. The patients were divided into two groups according to the semen sORP value. Sixty‐nine samples out of 393 showed a higher sORP value (>1.41 mv/10^6^/ml) and the rest samples showed a lower sORP value than 1.41 mv/10^6^/ml. The comparison of a transferable embryo and normal cleavage rate between high and low sORP was analyzed in 393 patients. Regarding fertilization rate, the data in 244 out of 393 patients who performed ICSI was utilized for fertilization rate comparison between high and low sORP groups. Regarding fertilization rate in conventional IVF, because of the number of control group was few, we could not investigate the influence.

**FIGURE 2:**
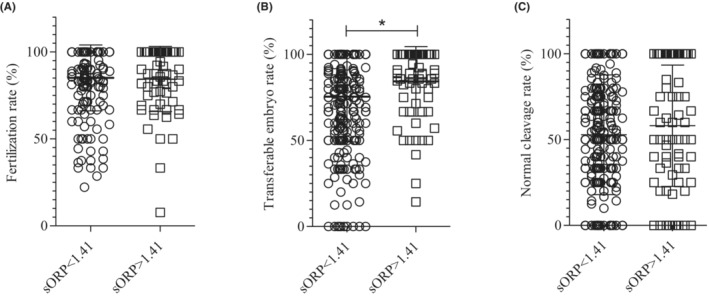
The embryology comparison in static oxidative reduction potential (sORP) cut‐off value is 1.41 (A) fertilization rate, (B) transferable embryo rate, (C) normal cleavage rate. The circle and square dot indicated the raw data. The bar center and both ends in the chart showed mean value and SD. The data of 244 patients who performed ICSI were included in (A) fertilization rate comparison, and 393 patients were done in (B) transferable embryo and (C) normal cleavage rate. Statistical significance was analyzed by *t*‐test.

**FIGURE A2:**
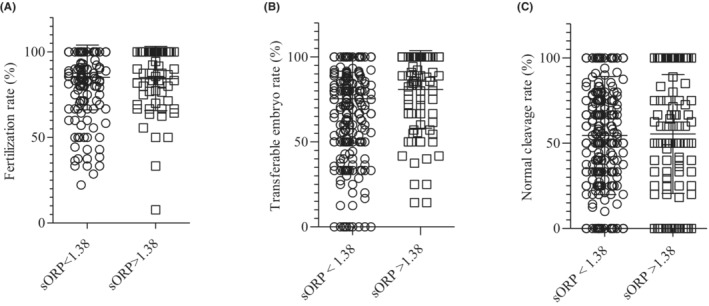
The embryology comparison in static oxidation‐reduction potential cut‐off value is 1.38. (A) fertilization rate, (B) transferable embryo rate, (C) normal cleavage rate. The circle and square dot indicated the raw data. The bar center and both ends in the chart showed mean value and SD. The data of 244 patients who performed ICSI were included in (A) fertilization rate comparison, and 393 patients were done in (B) transferable embryos and (C) normal cleavage rate. Statistical significance was analyzed by *t*‐test.

We predicted that semen samples with higher sORP value would be characterized as low fertilization and low embryo quality from previous data, However, in the group of higher semen, sORP did not show any decline of the fertilization rate, and normal cleavage rate, wherein we set the value as 1.38 mv/10^6^/ml (Figure A2) or 1.41 mv/10^6^/ml (Figure 2). And interestingly, the rate of transferable embryos was significantly increased in the group of semen showing higher sORP than 1.41 mv/10^6^/ml (Figure 2B).

### 3.4 Relationship between sORP value and the IVF outcomes

We have further analyzed the relationship between the sORP value, the clinical pregnancy rate, live birth rate, and abortion rate. Here, we anticipated seeing sORP has a negative correlation to the clinical pregnancy or live birth rate and a positive correlation with the abortion rate. However, unexpectedly, the 145 couples who were able to transfer embryos, have not shown any relationship between semen sORP value and these IVF outcomes (Tables 2 and 3). We believe these results suggest that analyzing the semen sORP may not be a predictive value for sperm quality and IVF outcomes.

**TABLE 2:** The comparison of clinical pregnancy, live birth, and abortion rate in static oxidation‐reduction potential cut‐off value is 1.41.

	sORP <1.41	sORP >1.41	*p*
Clinical pregnancy rate (%)	30.6 (37/121)	25.0 (6/24)	n.s.
Live birth rate (%)	5.0 (6/121)	0.0 (0/26)	n.s.
Abortion rate (%)	29.7 (11/37)	16.7 (1/6)	n.s.

*Note*: Clinical pregnancy and abortion rates (number) in lower and higher sORP were shown in each box. Statistical significance was analyzed by the chi‐square test.

Abbreviation: n.s., not siginificant.

**TABLE 3:** The relation static oxidation‐reduction potential vs clinical pregnancy, live birth, and abortion under logistic regression analysis.

	Odds ratio	CI (95%)	*p* Value	*N*
Clinical pregnancy	0.710	0.473–1.065	0.10	145
Live birth	0.998	0.929–1.073	0.97	145
Abortion	0.647	0.227–1.846	0.42	43

*Note*: Odds ratio and confidential interval (95%) were shown in boxes.

## 4 DISCUSSION

The present study's data showed that the oxidation–reduction potential (ORP) level in a male patient's semen correlated with his semen characteristics but not fertilization, embryo development, and pregnancy rate after IVF.

Oxidative stress is a common condition that can be found in all kinds of living cells due to unbalanced ROS regulation. And OS has adverse effects on the structural and functional integrity of sperm which is one of the major causes of defective sperm function and male infertility. The sperm DNA integrity is important in the dynamics of epigenetic reprogramming and the spermatozoa are one of the most vulnerable cells to OS which impairs active DNA demethylation as these cells have limited antioxidant defense mechanisms and a limited capacity for the detection and repair of DNA damage.^27^, ^28^ Both sperm mitochondrial and nuclear DNA could be damaged by oxidative stress conditions resulting in negative consequences on paternal genetic and epigenetic contribution to the development of an embryo and an increased disease burden in the offspring.^10^, ^13^, ^27^ Thus, the sperm DNA fragmentation index is a highly valuable index in male fertility evaluation.^29^, ^30^ And sperm preparation practices that are routinely followed in fertility clinics including ourselves, such as density gradients centrifugation and swim‐up procedures help to eliminate sperm with DNA fragmentation. However, these processes do not completely eliminate DNA fragmented sperm in the final processed sperms, and negative influences on IVF outcome have been observed frequently, though we did not investigate how extent the damaged sperm were excluded by semen preparation for IVF.^21^, ^31^, ^32^, ^33^ Therefore, we speculated that the level of original semen oxidative stress OS could be used as a predictive value for the male sperm quality and a good marker for predicting the fertilization rate, embryo development rate, the quality of the embryo, and the IVF outcomes.

Previous research indicated that the semen ORP level is closely associated with sperm DNA fragmentation, which could be a good marker to predict sperm functions and efficiency, though we did not investigate this relation in the present study.^11^, ^34^ The MiOXSYS system is recently introduced and getting popular in infertility clinics as it measures the semen ORP level and allows one to get an idea about the quality and the status of semen samples. In our study, despite the reference cut‐off values of MiOXSYS, the patient samples have been analyzed for the relationship between semen characteristics and the sORP values. Though most of the conventional semen parameters such as the patient's age, amount of white blood cells, total motile sperm count, the total volume of the sample, and abstinence, pH were not shown any significant correlation between the semen sORP, the total sperm concentration and sperm motility were shown a significant weak negative correlation with sORP value. In addition, there was a positive weak correlation between morphology and sORP. These results confirmed that our analytical parameters were technically feasible as they were comparable with the MiOXSYS system's guideline references.^7^, ^11^, ^18^

**FIGURE A3:**
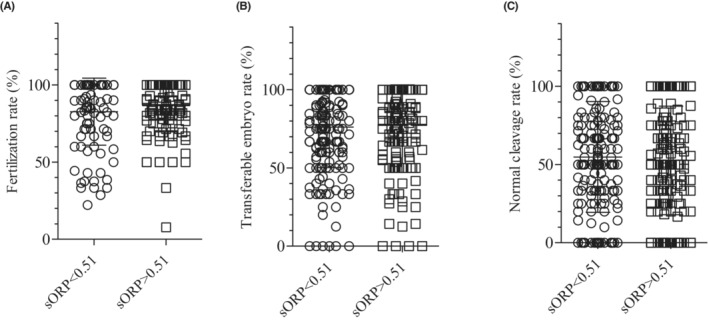
The embryology comparison in static oxidation‐reduction potential cut‐off value 0.51 (A) fertilization rate, (B) transferable embryo rate, (C) normal cleavage rate. The circle and square dot indicated the raw data. The bar center and both ends in the chart showed mean value and SD. The data of 244 patients who performed ICSI were included in (A) fertilization rate comparison, and 393 patients were done in (B) transferable embryo and (C) normal cleavage rate. Statistical significance was.analyzed by *t*‐test.

According to the theoretical concept of the MiOXSYS system, if the semen sORP level is higher than 1.41 mv/10^6^/ml, the sperm DNA fragmentation tends to be higher where fertilization capacity should be negatively affected. And along with that rate of fertilization rate, embryo development, quality of embryos, clinical pregnancy rate should also be negatively influenced. Interestingly, in our patient group, though the fertilization rate and cleavage range have not shown any significant difference, the semen with higher ORP has shown a significantly higher transferable embryo development rate compared to the low sORP group (Figure 2). Even the same tendency of developing higher quality embryos was found in the semen samples with higher sORP than 1.38 mv/10^6^/ml (Figure A2). However, we are unable to explain the exact molecular mechanism reason behind this tendency, which should be further studied in animal experiments. In addition, recently, Henkel et al have reported that the valid cut‐off value for semen sORP is 0.51 mv/10^6^/ml and when sperm is selected with this cut‐off value, it shows a clear correlation with IVF outcomes.^35^ We have recalculated the influence of sORP on IVF outcome on this new cut‐off value. Confirming the previous results, we could not see any significant relation between ORP value and IVF outcomes (Figure A3). This result suggested that the sORP value in the initial semen examination may not have a relation to IVF outcomes.

Moreover, we expected to see sORP has a negative correlation to the clinical pregnancy or live birth rate and a positive correlation with the abortion rate. However, with these patient samples, we could not see any relationship between sORP value and IVF outcomes (Tables 2 and 3). And further, though it was not significant, a lower abortion rate was found in the group with higher semen sORP value (Table 2). In contrast, paternal DNA is a key factor in fertilization and embryo development progression, paternal DNA fragmentation might not be a significant hurdle for embryo development and the pregnancy outcome.^36^ In the present study, we have carefully selected the female counterpart with their age and who has no oocyte defects or any other female fertility‐related factors. We believe that the results did not arise due to the female factors‐related phenomenon. Thus, according to our speculations, these results indirectly show that DNA fragmentation may not be only the factor that is altered with the oxidative stress in semen. And there might be some unknown, but supportive sperm factors that could be positively affected by the oxidative stress in individual semen. Therefore, it is still questionable whether measuring of sORP value is supportive to indicate the quality of sperm or decide the suitable infertility therapies. And further, an important point of semen examination is whether the result examination could predict IVF outcomes and the suitable approach of ART. Therefore, considering our results, we concluded that the level of semen sORP value in the initial semen examination is not sufficient to predict the fertilization capacity or quality of embryo development or IVF outcome. In addition, the rate of patients with abnormal sORP value was approximately 20% in this study. According to a previous publication, patients diagnosed with varicocele was higher sperm DNA fragmentation and sORP.^34^ Therefore, the negative relation between sORP and IVF outcomes might be observed in such paitients group. In this regard, the ORP data should be more extensively analyzed and validated in preclinical studies to get a clear idea about this relationship and IVF outcomes.

In conclusion, higher ROS‐mediated oxidation stress in semen and sperms are highly vulnerable to oxidative stress conditions. ORP can be measured as the level of oxidative stress in sperm samples and oxidation stress can negatively affect sperm DNA integrity. Some of the traditional sperm parameters are correlated with the semen ORP values. And controversially, sperm with higher ORP values are able to develop good embryos with a low abortion rate. However, the reason behind this phenomenon remains unknown and should further be studied and validated in animal studies. Even though it could explain DNA fragmentation by measuring ORP in semen, according to our clinical results there is no relationship with the IVF outcome which suggests that prediction of IVF outcome using sORP value in semen examination might not a feasible strategy to decide the IVF procedures and approaches.

## CONFLICT OF INTEREST

The authors declare no conflict of interest for this article.

## ETHICAL APPROVAL

This study was approved by Sunkaky medical corporation Ethical Review Board. All procedures followed were in accordance with the ethical standards of concerned institutional and national committees for human experimentation and the Helsinki Declaration of 1964 and its later amendments.

## INFORMED CONSENT

Informed consent was obtained from all patients for being included in the study.

## APPENDIX 1

**TABLE A1:** Female patient's background.

	Rate (%)	*N*
Tubal factor	18.6	80/430
Endometriosis	15.3	66/430
Thyroid dysfunction	20.7	89/430
Immunological infertility	14.9	64/430
Recurrent pregnancy loss	5.3	23/430
Hyperprolactinemia	5.8	25/430
Ovarian factor	21.9	94/430
Uterine fibroid	25.1	108/430
Polycystic ovary syndrome	4.0	17/430
Uterine adenomyosis	3.0	13/430
Secondary infertility	6.0	26/430

*Note*: The rate of each symptom: tubal factor, endometriosis, thyroid dysfunction, immunological infertility, recurrent pregnancy loss, hyperprolactinemia, ovarian factor, uterine fibroid, polycystic ovary syndrome, uterine adenomyosis and secondary infertility were shown.
